# Chicago Medical Society

**Published:** 1885-07

**Authors:** 


### SOCIETY REPORTS.

---

CHICAGO MEDICAL SOCIETY.

*Stated Meeting, June 15, 1885.*—The President, Professor C. T. PARKES, M. D., in the chair.

*A Successful Case of Nephrectomy.*—Dr. R. G. BOGUE reported an important case of nephrectomy. The patient was a woman, aged thirty-six, single, who had suffered from several attacks of acute rheumatism with cordiac complications, and also for nearly one year with a pus-discharging sore on one arm. About six years ago, while doing laundry work, she severely sprained her back, and this was followed by frequent and painful urination, the urine containing pus, blood and mucus. The pain in the back increased during menstruation. These symptoms continued until 1882, when a swelling, accompaned by pain and soreness, appeared in the right lumbar region. This swelling was finally opened and gave exit to a large quantity of pus, and remaining open, continued to discharge pus until the time of the operation; pus, blood and mucus at the same time appeared in the urine, which was voided with difficulty. At one time there was so much clotted blood in the bladder that it was only dislodged by breaking up the clot with a catheter and washing out the bladder. Later there was nearly a closure of the opening into the lumbar abscess, and a fluctuating tumor appeared near the old one, which was opened, and gave exit to a quantity of fœtid pus. From May 13 to August 27, 1884, there was free discharge of pus through the lumbar openings and the bladder. At no time was there a urinous odor to the discharge from the loin. Whenever the discharge from the loin was greater, pus appeared in less quantity in the urine, and vice versa.

In the belief that there was an abscess of the right kidney, on August 27, 1884, Dr. Bogue made an explanatory operation by making an opening into the loin, along the course of and just below the twelfth rib, including in its track the sinus. He came upon a dark, fluctuating mass, which upon puncture discharged a quantity of pus, and on further examination was found to be the capsule of the kidney, divided into compartments, and distended with pus. The sac was adherent to the surrounding tissues, and, upon separation, bled freely. The adhesions were quickly broken up and a stout ligature placed around the attachment or peduncle, which consisted of the ureter and renal blood-vessels. This was done by the aid of a large bent probe armed with silk. This ligature completely arrested the hemorrhage. Near this ligature the peduncle was transfixed by a double thread of strong silk and tied in two parts, the mass cut away, with ligatures left long and with drainage tube, that served to drain the cavity, which was cleansed with a carbolated solution and the wound dressed with oiled-silk, gauze and oakum. The patient convalesced rapidly with the exception of an attack of rheumatism and one rather free discharge of blood from the bladder, which followed traction on the ligatures. The ligatures did not separate until Dec. 7. But little pus came with the urine after the operation. There is yet a small fistulous opening in the track of the wound. The patient is now comparatively well. The mass removed was a distended kidney capsule with calices so distended that there was obliteration of the kidney tissue except at one point where there was enough to identify the structure.

Professor C. T. PARKES remarked that an interesting point in the report is that in which is described the method by which hemorrhage was arrested, as the greatest danger in this operation is from hemorrhage. Czernay had a case in which the hemorrhage was so severe that he ligated the aorta. Operations on the kidney are becoming quite frequent. In an interesting paper Dr. Gross gives his observations of over two hundred cases, and limits the cases in which an operation is suitable. Professor Parkes said that he wished to call the attention of the Society to the fact that there is sometimes an anomalous distribution of the arteries to the kidneys. He had met two cases in which the arteries entered the kidney at the lower end instead of at the hilus, and this should be remembered in cases of nephrectomy and especially nephrolithotomy. Sometimes the renal artery enters the kidney by two branches.

In answer to several questions by members of the society, Dr. Bogue replied that no vertical incision was necessary; that the diagnosis was positive only after the operation, which was an exploratory one; that the ureter and renal blood-vessels were ligated in mass, and that in a suspected case, where there was a well-defined, movable tumor in the abdomen, unaccompanied by pain, with pus in the urine, and difficult urination, which was relieved by washing out the bladder, if there was a reasonable probability that it was a suppurating kidney, he would advise an operation.

*The Continuous Curve Forceps.*—Dr. E. W. SAWYER read an interesting aud exhaustive paper on the Continued Pelvic Curve in the Obstetric Forceps, with remarks on forceps in general. He defined the perfect obstetric forceps to be an instrument with which it is practically impossible to injure the mother or fœtus, when skillfully used. In order that the forceps may be such a harmless extractor, after many years of observation and experience he had arrived at the following conclusions : First, that there should be a space of three and one-fourth inches between the compressing surfaces of the blades, when the inner surfaces of the handles are in contact. Any less space will fatally compress the fœtal head. The long diameter of the ellipse, bounded by the conjoined grasping curves of the blades, should be five and one-half inches, the distal opening of this ellipse, to correspond to the separation between the tips of the blades, may be three-fourths of an inch ; five and one-half inches from the blade tips, the opening should be two and one-half inches. If these measurements are less, severe injury may result to the fœtus. Second, in selecting an instrument, a straight-edged ruler should be placed at right angles to the long diameter of the blade, to determine if the center edge of the blade and the border of the fenestra are on the same plane, if not, such an instrument invariably injures the fœtal scalp. Third, the loose mortise joint lock, which allows a backward and forward motion of the handles, and thus enables the obstetrician to properly adjust the forceps to the fœtal head, is the only safe, simple and easily operated lock. Fourth, in compressing the fœtal head the closest imitation of nature is safest method to follow, hence a moment of compression during a pain should be followed by loosening the grasp of the head, which cannot be done with those instruments which permit the handles to be fixed together. Fifth, the length of the handles should be such as to allow the operator to reserve his force for difficult traction. The rounded space just in front of the lock, which admits of the introduction of a finger, is of special value as a point of traction when a great degree of compression is not demanded. Sixth, the blades of the instrument should be of the best steel, at least one-fourth of an inch square, at the shanks, and gradually thinned to about three thirty-seconds of an inch at the tips. Slighter material is so yielding as to permit the blades to be pulled off the head. Seventh, if an index be fixed to the fœtal head, as, for example, the handles of a forceps, in such a manner as to follow the direction given to the head by the unaided efforts of nature, an irregularly curved line would be described forward and upward, which would have its termination, at the moment of the escape of the head, at a point near the umbilicus of the woman. This curved line should be the guiding line to the attendant, who attempts to reinforce the expulsive force of the uterus with the forceps. Dr. Sawyer then exhibited a forceps of his own invention, in which the pelvic curve is extended to the ends of the handles, to guide the operator in producing traction in the direction of the curved line above mentioned. He claimed that in those cases where the occiput, or sinciput, presents posterior, and it is decided to deliver in this position, this curve will allow the operator to grasp the head with the forceps well forward over the ears, without its slipping over the occipital poles.

In answer to several objections as to the feasibility of securing a perfect forceps, Dr. Sawyer stated that he did not claim his forceps to be perfect, but that he had copied the best features of other forceps, and thinks he has an instrument which will do less injury to the mother or child than any other forceps with which he is acquainted. He believes it to be absolutely safe for the child, and was not intending to speak of the indications for using forceps, or the manner of their use, but that he had endeavored to incorporate in his paper some points about forceps in general, which he had never read or heard taught, and he hoped they would be useful to all who wished to approximate as nearly as possible the ideal forceps.

